# Enhancing User Experience Through User Study: Design of an mHealth Tool for Self-Management and Care Engagement of Cardiovascular Disease Patients

**DOI:** 10.2196/cardio.9000

**Published:** 2018-02-09

**Authors:** Hyunyoung Baek, Jung-Won Suh, Si-Hyuck Kang, Seungjin Kang, Tae Ho Lim, Hee Hwang, Sooyoung Yoo

**Affiliations:** ^1^ Healthcare Information and Communication Technology Research Center Office of eHealth Research and Businesses Seoul National University Bundang Hospital Seongnam Republic Of Korea; ^2^ Department of Cardiology, Seoul National University Bundang Hospital Seongnam Republic Of Korea; ^3^ HealthConnect Co, Ltd Seoul Republic Of Korea; ^4^ Department of Pediatrics, Seoul National University Bundang Hospital Seongnam Republic Of Korea

**Keywords:** cardiovascular disease, mHealth, mobile application, app, user-centered design

## Abstract

**Background:**

As patient communication, engagement, personal health data tracking, and up-to-date information became more efficient through mobile health (mHealth), cardiovascular diseases (CVD) and other diseases that require behavioral improvements in daily life are now capable of being managed and prevented more effectively. However, to increase patient engagement through mHealth, it is important for the initial design to consider functionality and usability factors and accurately assess user demands during the developmental process so that the app can be used continuously.

**Objective:**

The purpose of the study was to provide insightful information for developing mHealth service for patients with CVD based on user research to help enhance communication between patients and doctors.

**Methods:**

To drive the mobile functions and services needed to manage diseases in CVD patients, user research was conducted on patients and doctors at a tertiary general university hospital located in the Seoul metropolitan area of South Korea. Interviews and a survey were performed on patients (35 participants) and a focus group interview was conducted with doctors (5 participants). A mock-up mobile app was developed based on the user survey results, and a usability test was then conducted (8 participants) to identify factors that should be considered to improve usability.

**Results:**

The majority of patients showed a positive response in terms of their interest or intent to use an app for managing CVD. Functional features, such as communication with doctors, self-risk assessment, exercise, tailored education, blood pressure management, and health status recording had a score of 4.0 or higher on a 5-point Likert scale, showing that these functions were perceived to be useful to patients. The results of the mock-up usability test showed that inputting and visualizing blood pressure and other health conditions was required to be easier. The doctors requested a function that offered a comprehensive view of the patient’s daily health status by linking the mHealth app data with the hospital’s electronic health record system.

**Conclusions:**

Insights derived from a user study for developing an mHealth tool for CVD management, such as self-assessment and a communication channel between patients and doctors, may be helpful to improve patient engagement in care.

## Introduction

Cardiovascular disease (CVD) causes death and disabilities worldwide, with the number of deaths caused by this disease expected to reach 22.2 million by 2030 [1]. Among the risk factors of CVD, 80% pertain to smoking, drinking, and other unhealthy lifestyle habits [2]. Since the majority of these risk factors are preventable, having an accurate understanding of these factors and correcting one’s lifestyle is extremely important for preventing CVD [1]. Lifestyle behavior changes include regular daily exercise, eating healthy foods, abstaining from smoking, abstaining from alcohol, and taking medications in a set routine according to one’s prescriptions [3].

Mobile health (mHealth), which makes use of mobile phone apps and wearable devices, is growing rapidly in the health care industry. It offers patient communication, personal health tracking, and up-to-date information regardless of the time and place. Through continuous guidance on health activities, it is anticipated that health can be increased with little financial burden or effort. This can improve patients’ health-related activities or conditions to ultimately prevent their health from worsening or prevent further diseases. It may also be effective in preventing and managing chronic diseases such as CVD, for which self-care through improving one’s behaviors is an important factor.

According to study results of the American Heart Association, in April 2015 there were 12,991 apps on iTunes and 1420 apps on Google Play about weight management, exercise, smoking cessation, diabetes control, blood pressure management, cholesterol management, and medication management through mHealth for CVD prevention [4]. This shows that many apps for CVD are already being developed and researched. However, whether or not these apps are being actively used is as yet unknown. Many mobile programs have low utilization rates [5], and surveys show it is difficult for most mobile apps to exceed 3 months’ of use after the initial download [6,7].

To increase patient engagement with mHealth, apps must be designed from the user’s point of view so that they can be used effectively. Apps with a user-centered design have high usability and lower risk of failure, reduced costs from a long-term perspective, and improved overall quality [8]. The provision of evidence-based customized content that reflects the needs of the patient is also a very important factor.

Hence, this study aimed to develop a customized mHealth service optimized for patients with CVD through user research and usability testing. The purpose of the study was to provide information for developing an mHealth tool with features that support health connections between patients and doctors, and to improve the usability of such a tool.

## Methods

**Figure 1:**
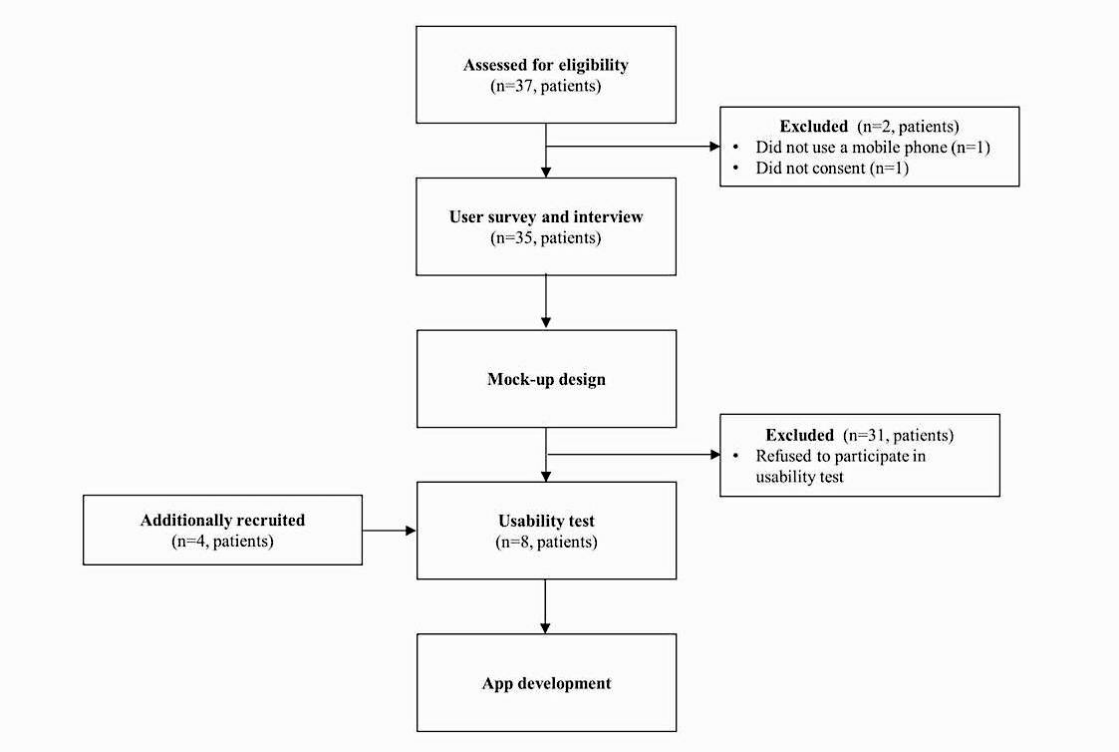
Study overview.

To develop an mHealth tool targeting patients with CVD, three types of user research and user experience investigations including surveys and interviews with patients, focus group interviews with doctors, and a usability test were conducted (Figure 1).

This study was approved by the Institutional Review Board of Seoul National University Bundang Hospital, and individuals who consented to take part in this study became the research participants (IRB No: B-1612-373-308).

### Survey and Interview With Patients With Cardiovascular Disease

To enable sustainable use of a mobile service, characteristics of users must be assessed and their demands and needs accurately understood [9]. To this end, user research was conducted through face-to-face interviews and a survey targeting patients with CVD.

The survey questionnaire was drafted based on a survey from existing mHealth-related literature [10,11], and the final version was completed after review and discussion by a group of experts, including two doctors from a cardiology department, one medical informatics professor, one nurse, two researchers, and three developers. The survey questions were divided into the three major categories of participant demographics (4 questions), current health care status (8 questions), and health app perceptions and demands (16 questions), for a total of 28 questions.

Study enrollment targeted outpatients and inpatients from the cardiology department of a tertiary general university hospital located in the Seoul metropolitan area of South Korea. The inclusion criteria were patients with CVD aged 30 years or older with their own mobile phone. The exclusion criteria were those with psychiatric disabilities, such as delirium, or diagnosed with unrelated CVD that was not covered by the high-risk and low-risk groups of CVD disease. Patients were classified as high risk if they had received a diagnosis of unstable angina, non-ST-elevation myocardial infarction, ST-elevation myocardial infarction, myocardial infarction, coronary disease, coronary atherosclerosis, angina pectoris, or acute myocardial infarction. Patients were classified as low risk if they had received a diagnosis of atrial fibrillation, hypertension, chest pain, vasovagal syncope, variant angina, hypertension, and dyspnea on exertion.

The survey period spanned 17 days from January 9 to 25, 2017; a structured self-reported survey and fact-to-face interviews were conducted with 35 of 37 patients after excluding two patients who did not use a mobile phone or who did not consent to the study.

The survey and interview was conducted by two researchers. When a patient agreed to participate, a researcher first provided a thorough explanation of the study overview, survey method, and privacy and security policies regarding collected data before conducting the survey with the patient. After finishing the survey, an open-ended interview was conducted. Examples of the interview questions include:

1. What signs and symptoms of cardiovascular disease have you ever had?
2. How did you manage your health before the symptoms appeared?
3. How have you managed your health since you developed the disease?
4. What barriers or difficulties exist when managing your disease?
5. What do you think you need more of to manage your disease effectively?

In addition, participants were free to speak about the expected effects of mHealth tools for CVD. Various experiences and perspectives on disease management were collected from participants in an informal atmosphere. During the user research, one researcher led the survey and interview, and the other documented all questions, answers, and opinions from participants [12]. The interview was audio-recorded and fully transcribed by the interviewer. Each transcript was supplemented and compared with the interview notes to ensure its accuracy

The interview transcripts were analyzed using the constant comparative method [13]. First, two researchers conducted open coding of the transcripts to identify distinct opinions. After the initial coding, researchers continuously and repeatedly reviewed the transcripts to group or regroup concepts to determine users’ requirements of an mHealth tool for cardiovascular patients.

The data collected from the survey were analyzed using the R 3.3.1 software version.

### Focus Group Interview With Cardiologists

The focus group interview method is a qualitative data collection technique used to obtain detailed information on the thoughts, emotions, attitudes, or experiences of participants. It is a suitable model for sharing patient care experiences [14,15]. The purpose of this part of the study was to conduct a focus group interview with doctors from the cardiology department to derive needs and demands regarding mobile services based on their experiences of daily clinical practice. We asked five cardiologists to participate in the focus group interview and they all agreed to do so.

The focus group interview lasted approximately 90 minutes and was moderated by a user research expert. After introducing the research overview, the moderator provided five discussion topics, including criteria on target patients as end users of the cardiovascular mHealth tool, available assessment tools, available questionnaires, education materials useful for personalized education, and mHealth app functions useful for managing cardiovascular disease. Each topic was freely discussed for 15 minutes. Additionally, doctors were asked to provide a layout sketch for a doctors’ Web screen that would be linked with the hospital’s electronic health record (EHR) system and the mHealth app. All sessions were audio-recorded and all field notes of participating coresearchers were collected.

**Table 1:** Scenarios of the usability test on the mHealth cardiovascular disease mock-up app.

Task	Task item	Test activity
1	Log-in	Logging in to the app using a given test user account
2	My health	Checking one’s comprehensive health status
3	Daily mission	Checking one’s daily mission, as prescribed by a physician
4	Health information	Finding the health information one wants, which can be tailored to one’s disease
5	Health questionnaire	Sounding an alarm for a new questionnaire survey after which participants try to fill in
6	Self-management (blood pressure, blood sugar test, body weight)	Inputting a given blood pressure value (eg, 120/80) into the app, then viewing the blood pressure trend via a graph
7	Diary	Choosing an appropriate icon based on one’s daily moods and symptoms

Two researchers analyzed the opinions from doctors using a card-sorting method [16] to derive key functions for the cardiovascular mHealth tool. Researchers extracted meaningful sentences or words from the recorded interviews and categorized them into groups. These groups were each given a topic. Doctors were interviewed on each of these topics and their responses were analyzed.

### Usability Test With Patients With Cardiovascular Disease

The usability test is a method to evaluate how easily the end user understands, learns, and uses software or an app under specific conditions [17]. The purpose at this stage of the study was to (1) test the usability of cardiovascular care apps developed through prototypes and (2) develop an actual app based on user experiences and needs that were derived through testing.

This study conducted a usability test on an app that would be offered to patients based on functions derived through a literature review and the aforementioned user research and focus group interview with patients and doctors, respectively. A mobile phone app designed using a mock-up tool was used as the test tool. Mock-ups are visualizations that enable users to experience the functions that will be implemented in an app [18,19]. For this study, a free HTML5-based Web app prototyping online tool was used to create a user interface that was similar to the actual app screen that would be developed. The usability test was conducted with actual users through an Android mobile phone.

The test was conducted over 2 weeks from March 6 to 20, 2017, with eight participants, including four patients with CVD among the user survey participants and four patients with CVD who were additionally recruited voluntarily through poster advertisements in the hospital. Although the usability test was offered to all participants in the initial user survey, only four agreed to participate in the test. The inclusion and exclusion criteria for the usability test were the same as those for the survey and interview. The test was conducted in a separate space. A researcher provided the mock-up app for the participants and observed and recorded whether they were able to proceed with the given test scenario on their own.

The test scenario involved having the participant directly inquire about and enter information according to a given task (Table 1) by focusing on the main menu (log-in, my health, daily mission, health information, health questionnaire, self-management, diary), and intervention by the test administrator was minimized during testing. A face-to-face interview about the first impression of the app, overall satisfaction, service inconveniences, and improvements followed thereafter.

## Results

### Survey and Interview Demographics

Of the 35 survey participants who were patients, there were more males (n=28, 80%) than females (n=7, 20%). Regarding age, 17 were younger than 60 years (49%) and 18 were 60 years or older (51%). The majority of patients were outpatients of the cardiology department (31/35, 89%), 18 of whom (51%) were in the high-risk group and 17 of whom (49%) were in the low-risk group based on their diagnoses. Fourteen participants (40%) had comorbidity, seven had diabetes, and six had hyperlipidemia. All participants owned a mobile phone, and most were Android users (34/35, 97%) (Table 2).

### Patients’ Perceptions of Health Management

**Figure 2:**
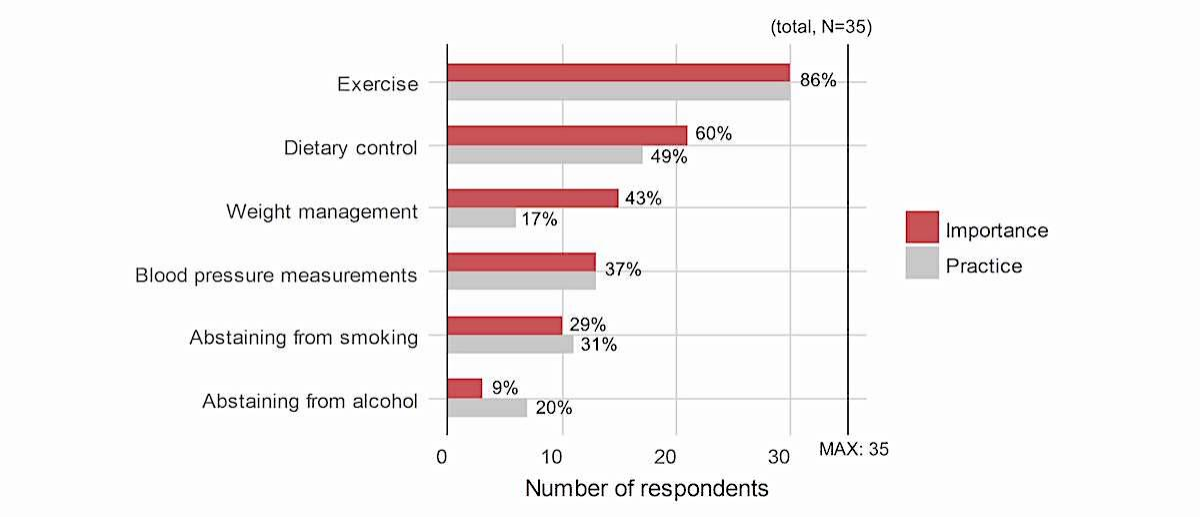
Comparison between patients’ perceptions of importance and actual practice regarding their management of cardiovascular disease.

Of the six items (exercise, dietary control, blood pressure measurements, abstaining from smoking, abstaining from alcohol, and weight management) that were previously surveyed to be important for CVD prevention and care in existing literature [20], patients were asked to select three items that they believed were important and three items that they practiced. The results are shown in Figure 2.

The top three items that patients thought were important included exercise, dietary control, and weight management, and the items that they practiced included exercise, dietary control, and measuring blood pressure. Many participants selected exercise and dietary control as both important items and items they practiced, whereas weight management was found to be an item that patients believed was important but had difficulty practicing.

### Patients’ Perceptions of the Use of Mobile Apps for Health Management

**Figure 3:**
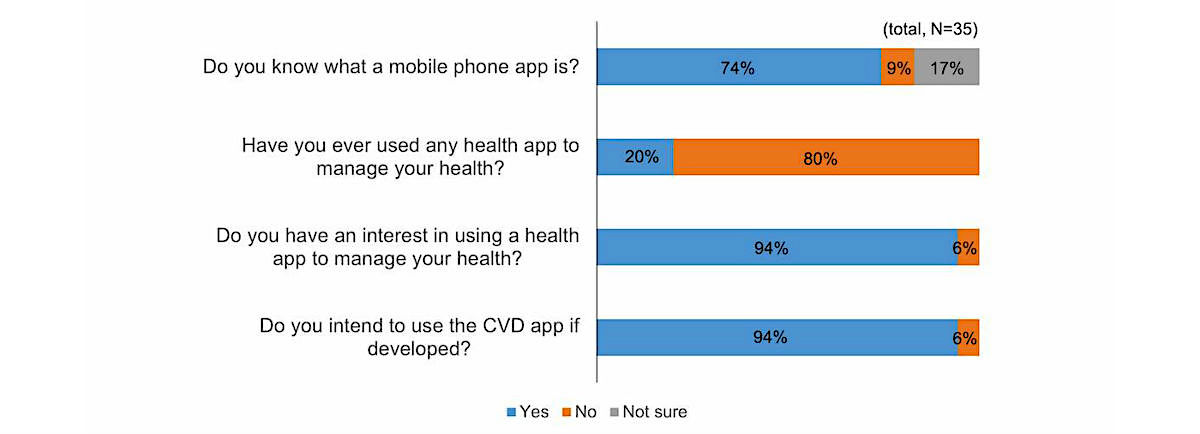
Perceptions of the use of mobile apps for health management.

According to the survey results on awareness regarding apps among the 35 participants, 74% (26/35) were aware of mobile phone apps, but only 20% (7/35) had experience using a health care app. However, the majority of participants (94%, 33/35) responded positively that they were interested and would be willing to use apps for managing CVD (Figure 3).

**Table 2:** Demographic information of patients who participated in the survey and interview (N=35).

Variables		n (%)
**Gender**		
	Male	28 (80)
	Female	7 (20)
**Age (years)**		
	<60	17 (49)
	≥60	18 (51)
**Number of years visited hospitals**		
	<3 years	20 (57)
	≥3 years	15 (43)
**Treatment type**		
	Inpatient	4 (11)
	Outpatient	31 (89)
**Risk group**		
	High risk	18 (51)
	Low risk	17 (49)
**Comorbidity**		
	Yes	14 (40)
	No	21 (60)
**Mobile device type**		
	Android	34 (97)
	iOS	1 (3)

### Patients’ Perceptions of Useful Functions of Apps for Cardiovascular Disease

**Figure 4:**
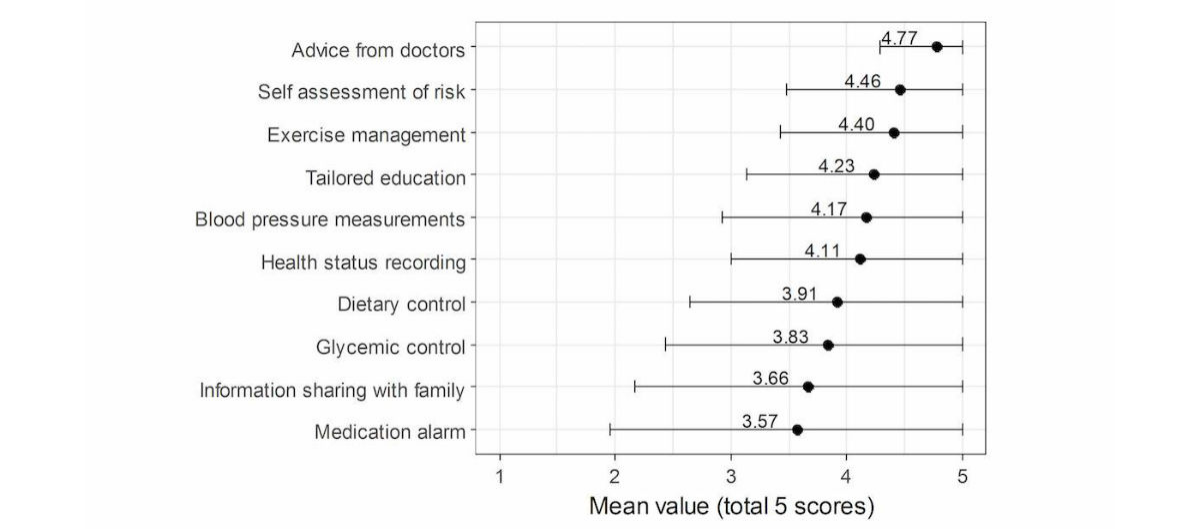
Patients’ perception of the usefulness of various functions of cardiovascular disease mobile apps (error bars represent standard deviation).

On surveying the usefulness of 10 different functions to be implemented in the CVD app on a 5-point scale, there were positive reactions to all functions, with scores of 3.5 points or higher on all. Advice from doctors was highest with a mean score of 4.77 (SD 0.49), followed by self-assessment of risk with mean 4.46 (SD 0.98), and exercise management with mean 4.40 (SD 0.98). The medication alarm function had the lowest score of mean 3.57 (SD 1.61) (Figure 4).

### Other Patients’ Interview Results

During the survey, face-to-face interviews were also conducted with each participant, who provided three to four different opinions on the requirements of the mHealth tool. The most frequently and commonly required features were (1) easy app control and use, (2) up-to-date information on disease and health, (3) management of presymptoms of CVD and self-assessment, (4) check of current health status, and (5) communication with doctors.

### Focus Group Interview Results With Cardiologists

Based on the results of the interview conducted with doctors from the cardiology department, key functions of the app that would be provided to their patients were identified. The cardiologists indicated that personal health data involved in self-care for managing CVD, such as blood pressure, blood glucose, and step count, must be linked to various devices, in addition to the manual entry function, for the user’s convenience and data accuracy. Diet information is extremely important for managing CVD; however, because it is cumbersome and difficult to consistently collect through an app, this service was suggested to be excluded.

Various health questionnaires are utilized during treatment; therefore, a health questionnaire feature was recommended to be implemented in the app. This would improve the effectiveness of treatment by allowing patients to complete questionnaire items in advance before consultations. However, the health questionnaire period must be set to an appropriate time interval so that patients do not receive too many alerts. In addition, because personal/family events are important regarding patient health status, a memo function was requested to allow patients to record daily or unexpected events.

Beyond the app provided to patients, there was also a need for an app through which doctors can view patients’ behavioral data. This must be linked with the hospital’s EHR system so that patients’ entries into the app can be viewed simultaneously with EHR clinical data.

**Figure 5:**
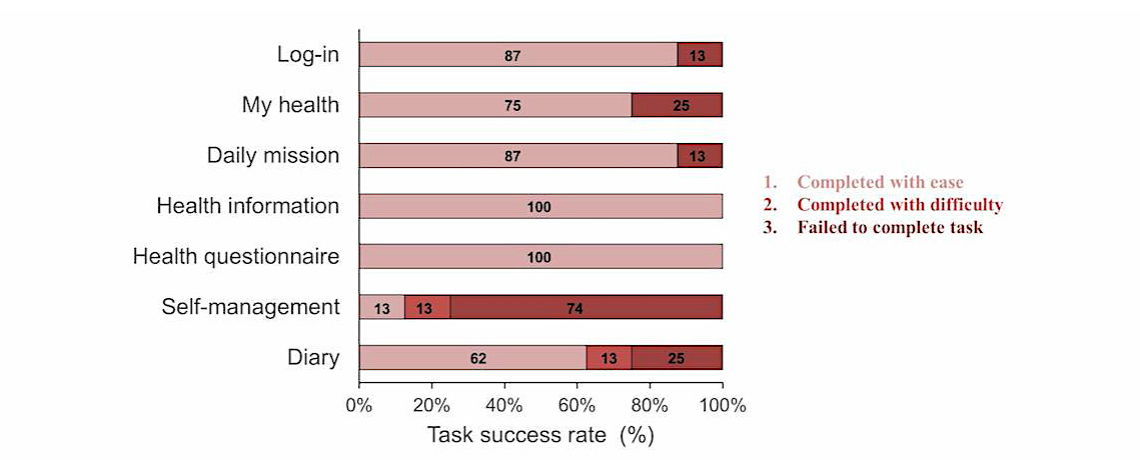
Participants’ success rate using the app by task (n=8).

**Table 3:** Functions and issues/implications of the mock-up design of the user interface for a cardiovascular disease app. EHR: electronic health record.

Menu	Function	Issues	Implications
My health	Framingham risk score [21]; laboratory test results (triglycerides, low-density and high-density lipoprotein cholesterol, and glycated hemoglobin A_1c_)	Uncertain what the score means	A detailed description of the risk score must be provided
Daily mission	Target values of body weight, blood sugar, and steps, as prescribed by the physician; my health status	Medical terms are difficult to understand	Medical terms used should be changed to simple, easy-to-understand terms for elderly patients
Health information	Education materials (PDF file, video) [22,23]; scope: hypertension, myocardial infarction, cardiac rehabilitation, angina pectoris	The users want personalized health information	Medical education materials should be tailored according to the patients’ diseases and conditions
Health questionnaire	Various questionnaires; basic questionnaire: anthropometry (height, body weight), blood pressure, smoking, hypertension drugs, diabetes, family history; outpatient questionnaire: drug compliance, dietary supplement, Canadian Cardiovascular Society [24], New York Heart Association [25], edema assessment; periodic questionnaire: depression, Patient Health Questionnaire (PHQ-2) [26]	Recognition of click icon buttons and their images is low; alarm fatigue is a concern	Easy-to-understand icon designs are needed; alarms for questionnaires should be minimized or set by individual users
Self-management	Self-management (device interface or manual input); items: body weight, blood pressure, blood glucose test, steps; trend view of health status via graph	The input button is hard to find and the recognition of the graph value is difficult	Easy-to-understand icon design and a detailed description of graph value are needed
Diary	Daily mood and symptoms (view by week and month); write memos on recent events	The icon image is not clear; it is hoped that the input data are shared with physician	Overall design of icons should be improved; any data input by the patient must/should appear in the physician’s EHR

### Usability Test Results

Of the eight patients who participated in the usability test on the mock-up mobile app, six were male and two were female. Seven participants were younger than 60 years and one was 60 years or older. Their mean age was 52.5 (SD 10.5) years.

Regarding the success rate of a given task, results were divided into three types (completed with ease, completed with difficulty, failed). In terms of the categorization, “completed with ease” reflected when the participant was able to complete tasks on their own by looking at the scenario; “completed with difficulty” was when the participant was unable to succeed on his/her own, but completed the tasks after receiving help from the researcher; and “failed” was when the participant was unable to complete the task or understand despite the researcher’s help. The success rate was 100% for tasks 4 (health information) and 5 (health questionnaire), and most other tasks had a success rate of at least 70%. This showed that users were able to use the app with relative ease. But the success rate for task 6 (self-management) was 25% and most participants failed. This shows that this menu user interface (UI) required overall improvement (Figure 5).

**Figure 6:**
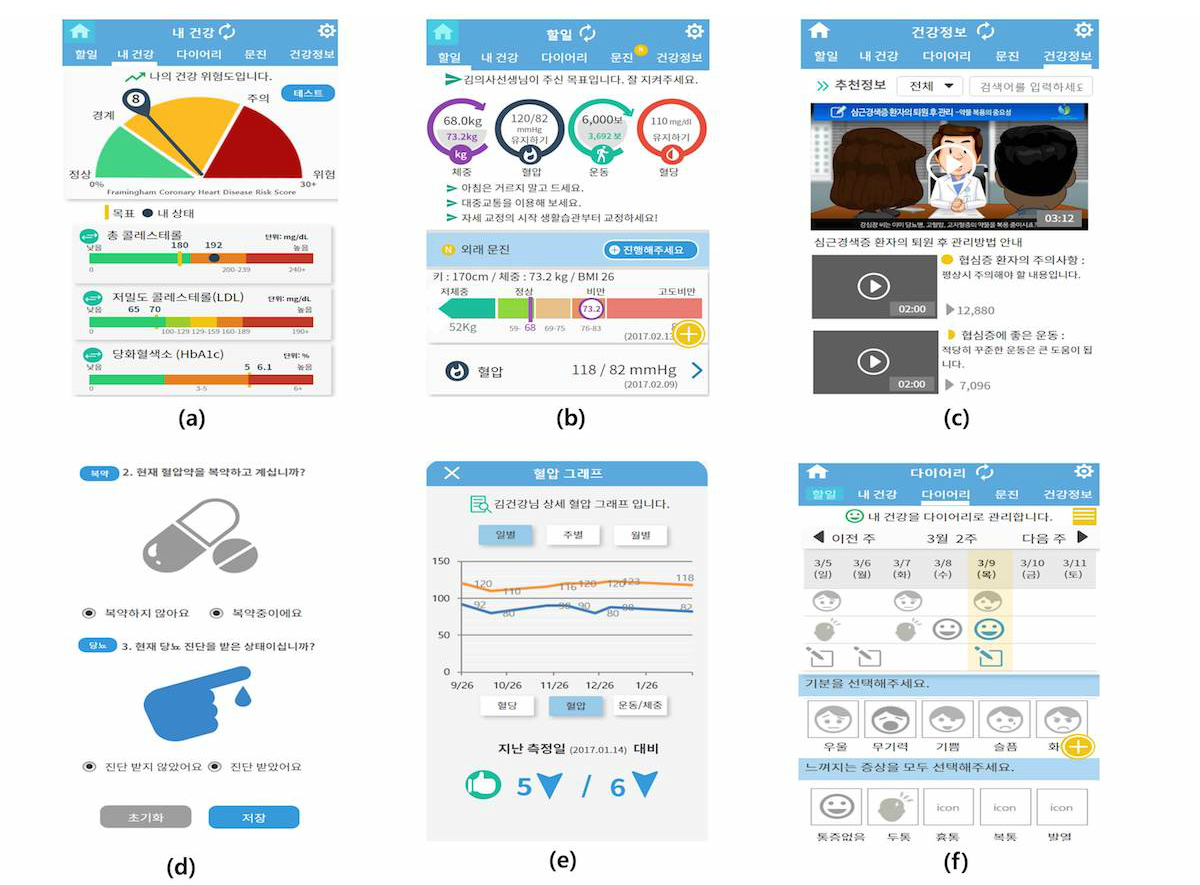
User interfaces of the mock-up design for a cardiovascular disease app: (a) my health, (b) daily mission, (c) health information, (d) health questionnaire, (e) self-management, and (f) diary.

Regarding the participants’ satisfaction with the app, the score was mean 3.6 (SD 1.2) out of 5 points, which shows that they were somewhat satisfied with the overall design or function. They responded that this mHealth tool would be very useful for patients who did not know or have an accurate understanding of how to manage CVD. They also anticipated that they would be able to manage their disease more efficiently if the data that they normally recorded manually on a daily basis could be easily entered in the app or linked and shared with their doctors. However, there were also issues and inconveniences for each menu that were identified through the usability test.

Table 3 shows the issues and matters for improvement that were derived from the usability test with the UI (Figure 6) and functions defined through literature review, user study, and focus group interviews. There were many requests for additional explanations on the information provided and to improve the image icon so that it could be seen intuitively.

## Discussion

### Principal Findings

Many mHealth programs are already being developed and actively researched to prevent and manage CVDs. It has been reported that when patients with CVD use digital tools as mediators there are positive effects such as improved outcomes, which can result in a reduction of risk factors that lead to CVD [27,28]. However, to maintain positive outcomes, it is important to design a model that can make the program sustainable. To this end, characteristics of end users of the program must first be accurately assessed. Users must also directly participate from the initial development stage, so that the program can be developed with a design and content that reflects their opinions.

This study designed an mHealth tool for patients with CVD from the perspective of actual users including both patients and doctors through the user study and usability testing. The majority of users were 60 years of age or older and 40% of the participants had comorbidity such as diabetes or hyperlipidemia. Most participants had very little experience with using a health care mobile app; however, their interest or intent to use an app for managing CVD was high, despite the fact that most were seniors who were unfamiliar with the information technology environment. In previous studies, there were many elderly users aged 65 years or older within the mHealth domain for CVD care [29]. Although the ripple effect was extremely low in terms of app use because it was difficult for them to adapt to technology, their interest or intent to participate was still reported to be very high [27,28].

This implies that a CVD management program that utilizes mHealth must be developed based on such high user interest and intent. However, additional supplementation is required for these programs to be used effectively. To this end, characteristics of elderly users, who are the majority of this demographic, must be sufficiently reflected and the program must be supported by profound evidence-based content so that comorbidity can be managed. One of the reasons why many health-related apps fail is because they lack evidence-based content [30-33].

Evidence-based content for CVD management programs includes known CVD risk factors through previous research results [34], the majority of which are behavioral factors that can be modified from an individual’s lifestyle. The seven behaviors, which are known as Life’s Simple 7 recommended by the American Health Association, include sufficient exercise, healthy eating habits, abstaining from smoking, maintaining an appropriate body mass index, and managing blood pressure, cholesterol, and blood glucose at normal levels. If this type of healthy lifestyle and health figures can be maintained, the mortality rate from cardiac diseases could potentially decrease by up to 20% by the year 2020, owing to a state of “ideal cardiovascular health” [20]. In this study, an app was created to reflect the requirements of users during content creation so as to make it an easy-to-use tool that offered updated information and enabled self-assessment [21]. Moreover, because advice from doctors or communication with them, which received the highest score from the perspective of usability out of all the app functions, is an important factor in a successful mHealth model [35], a Web portal that is visible to medical professionals during treatment should be also developed so that patient-reported health data can be shared and a bidirectional service model can be implemented.

During the focus group interview with doctors, one requested that a health questionnaire service related to CVD be included so that doctors could refer to these data during consultations. A health questionnaire that can assess depression was included, in which patients inputted their daily mood and symptoms so that their doctors could immediately assess their mood and changes during treatment and take necessary action. Depression is reported to be a critical factor that increases the risk and mortality rate of CVD [36]. However, because it is a difficult disorder to assess during short consultation times, if the patient’s mood can be assessed through mHealth and revealed to the medical professionals, this will be helpful for the patient’s course of treatment.

User-centered design is an important matter of consideration in addition to content while developing a service. According to surveys from existing studies, apps that manage chronic diseases must have user-centered design features, including visualizing patient-related trends through a graph, sending alerts when care or follow-up is required, and urging users to continuously manage their conditions through communication functions such as text messages. Providing visual educational materials using videos, proposing tailored goals, accessing patients’ health record data, and including functions that enable patients to record their status were also mentioned as important factors of user-centered design features [37]. This study developed an app reflecting all these factors, and improved the UI and naming for some menus based on issues that were identified through the usability test.

Going forward, app usability must be evaluated in an actual clinical environment through clinical testing using an app that has been developed based on the results of this study, and its potential and usability as a sustainable model must also be studied. Further, an effective way to input diet information in a mobile environment should also be considered. The clinical effectiveness of the CVD mHealth tool will also be evaluated in the future.

### Limitations of the Study

A limitation of this study was that the patient’s diet, which was found to be an important item for managing CVD in previous literature reviews and user research, was excluded. Because each individual’s eating habits differ and there is a large amount of data that must be entered, we followed the results of the focus group interview discussion that this function would make the app cumbersome to use. Additionally, because the usability test was conducted through an app that was created using a mock-up tool, there may be differences in usability compared to that of the actual app that is developed.

### Conclusions

This study developed an mHealth tool that can effectively manage CVDs through user research that involved the direct participation of doctors from the cardiology department and patients with CVD. The field of mHealth may most effectively mediate an individual’s health behaviors. For diseases such as CVD that are highly influenced by various lifestyle habits and chronic diseases, the provision of a program that uses mHealth may greatly assist in the management and prevention of diseases.

There is a demand for an mHealth tool to include functions that effectively support communication between patients and doctors, self-assessment and evaluation of risk, and exercise management, all which were deduced through user research in this study with respect to CVD management. The functional features should be provided to patients with high usability to ensure sustainable use of mHealth tools and enhance patient engagement in the medical environment.

## Abbreviations

CVD: cardiovascular disease
EHR: electronic health record
mHealth: mobile health
UI: user interface

## References

[1] Kelli HM, Witbrodt B, Shah A (2017). The Future of Mobile Health Applications and Devices in Cardiovascular Health. Euro Med J Innov.

[2] Pfaeffli DL, Dobson R, Whittaker R, Maddison R (2016). The effectiveness of mobile-health behaviour change interventions for cardiovascular disease self-management: A systematic review. Eur J Prev Cardiol.

[3] Piepoli MF, Corrà U, Adamopoulos S, Benzer W, Bjarnason-Wehrens B, Cupples M, Dendale P, Doherty P, Gaita D, Höfer S, McGee H, Mendes M, Niebauer J, Pogosova N, Garcia-Porrero E, Rauch B, Schmid JP, Giannuzzi P (2014). Secondary prevention in the clinical management of patients with cardiovascular diseases. Core components, standards and outcome measures for referral and delivery: a policy statement from the cardiac rehabilitation section of the European Association for Cardiovascular Prevention & Rehabilitation. Endorsed by the Committee for Practice Guidelines of the European Society of Cardiology. Eur J Prev Cardiol.

[4] Burke LE, Ma J, Azar KM, Bennett GG, Peterson ED, Zheng Y, Riley W, Stephens J, Shah SH, Suffoletto B, Turan TN, Spring B, Steinberger J, Quinn CC, American Heart Association Publications Committee of the Council on Epidemiology and Prevention, Behavior Change Committee of the Council on Cardiometabolic Health, Council on Cardiovascular and Stroke Nursing, Council on Functional Genomics and Translational Biology, Council on Quality of Care and Outcomes Research, Stroke Council (2015). Current science on consumer use of mobile health for cardiovascular disease prevention: a scientific statement from the American Heart Association. Circulation.

[5] Eysenbach G (2005). The law of attrition. J Med Internet Res.

[6] Furner CP, Racherla P, Babb JS (2014). Mobile app stickiness (MASS) and mobile interactivity: a conceptual model. Mark Rev.

[7] Krebs P, Duncan DT (2015). Health app use among US mobile phone owners: a national survey. JMIR Mhealth Uhealth.

[8] Humayoun S, Dubinsky Y, Catarci T (2011). A three-fold integration framework to incorporate user-centered design into agile software development. Human Centered Design.

[9] Hancock J, Shemie SD, Lotherington K, Appleby A, Hall R (2017). Development of a Canadian deceased donation education program for health professionals: a needs assessment survey. Can J Anaesth.

[10] Ramirez V, Johnson E, Gonzalez C, Ramirez V, Rubino B, Rossetti G (2016). Assessing the use of mobile health technology by patients: an observational study in primary care clinics. JMIR Mhealth Uhealth.

[11] Schuuring MJ, Backx AP, Zwart R, Veelenturf AH, Robbers-Visser D, Groenink M, Abu-Hanna A, Bruining N, Schijven MP, Mulder BJ, Bouma BJ (2016). Mobile health in adults with congenital heart disease: current use and future needs. Neth Heart J.

[12] Anderson J, Aydin C (2005). Evaluating the Organizational Impact of Health Care Information System.

[13] Glaser B, Strauss AL (1999). Discovery of Grounded Theory: Strategies for Qualitative Research.

[14] Holloway I, Galvin K (2016). Qualitative Research in Nursing and Healthcare.

[15] Hamnes B, Rønningen A, Skarbø A (2017). Experiences of participating in return-to-work group programmes for people with musculoskeletal disorders: A focus group study. Musculoskeletal Care.

[16] Hendry M, Pasterfield D, Gollins S, Adams R, Evans M, Fiander A, Robling M, Campbell C, Bekkers M, Hiscock J, Nafees S, Rose J, Stanley M, Williams O, Makin M, Wilkinson C (2017). Talking about human papillomavirus and cancer: development of consultation guides through lay and professional stakeholder coproduction using qualitative, quantitative and secondary data. BMJ Open.

[17] Tiburcio M, Lara MA, Aguilar AA, Fernández M, Martínez VN, Sánchez A (2016). Web-based intervention to reduce substance abuse and depressive symptoms in Mexico: development and usability test. JMIR Ment Health.

[18] Wang F, Hannafin MJ (2005). Design-based research and technology-enhanced learning environments. Educ Technol Res Dev.

[19] Mann DM, Quintiliani LM, Reddy S, Kitos NR, Weng M (2014). Dietary approaches to stop hypertension: lessons learned from a case study on the development of an mHealth behavior change system. JMIR Mhealth Uhealth.

[20] Lloyd-Jones DM, Hong Y, Labarthe D, Mozaffarian D, Appel LJ, Van HL, Greenlund K, Daniels S, Nichol G, Tomaselli GF, Arnett DK, Fonarow GC, Ho PM, Lauer MS, Masoudi FA, Robertson RM, Roger V, Schwamm LH, Sorlie P, Yancy CW, Rosamond WD, American Heart Association Strategic Planning Task Force and Statistics Committee (2010). Defining and setting national goals for cardiovascular health promotion and disease reduction: the American Heart Association's strategic Impact Goal through 2020 and beyond. Circulation.

[21] Kannel W, Wilson P (2016). Risk factors for cardiovascular disease and the Framingham Study Equation. Comprehensive Management of High Risk Cardiovascular Patients.

[22] Rumsfeld JS, Brooks SC, Aufderheide TP, Leary M, Bradley SM, Nkonde-Price C, Schwamm LH, Jessup M, Ferrer JME, Merchant RM, American Heart Association Emergency Cardiovascular Care Committee, Council on Cardiopulmonary‚ Critical Care‚ Perioperative Resuscitation, Council on Quality of Care and Outcomes Research, Council on Cardiovascular and Stroke Nursing, Council on Epidemiology and Prevention (2016). Use of mobile devices, social media, and crowdsourcing as digital strategies to improve emergency cardiovascular care: a scientific statement from the American Heart Association. Circulation.

[23] Cho MJ, Sim JL, Hwang SY (2014). Development of smartphone educational application for patients with coronary artery disease. Healthc Inform Res.

[24] Bress A, Dodson J, Sauer B, Patterson O, Alba P, Duvall S (2016). Canadian Cardiovascular Society (CCS) angina classification extracted from clinical notes by natural language processing: validation and association with healthcare utilization in an integrated health.

[25] McMurray J, Gong J, Rouleau J, Solomon S, Swedberg K, Zile M (2016). Efficacy and safety of Sacubitril/Valsartan in patients in NYHA functional class IV. An analysis of PARADIGM-HF. Circulation.

[26] Thibault JM, Steiner RW (2004). Efficient identification of adults with depression and dementia. Am Fam Physician.

[27] Widmer RJ, Collins NM, Collins CS, West CP, Lerman LO, Lerman A (2015). Digital health interventions for the prevention of cardiovascular disease: a systematic review and meta-analysis. Mayo Clin Proc.

[28] Hamine S, Gerth-Guyette E, Faulx D, Green BB, Ginsburg AS (2015). Impact of mHealth chronic disease management on treatment adherence and patient outcomes: a systematic review. J Med Internet Res.

[29] Smith A (2014). Pew Research Center Internet & Technology.

[30] Breton ER, Fuemmeler BF, Abroms LC (2011). Weight loss-there is an app for that! But does it adhere to evidence-informed practices?. Transl Behav Med.

[31] Azar KM, Lesser LI, Laing BY, Stephens J, Aurora MS, Burke LE, Palaniappan LP (2013). Mobile applications for weight management: theory-based content analysis. Am J Prev Med.

[32] Petakov A, Nesmith K (2014). Weight loss using evidence-based strategies in mobile apps. Am J Prev Med.

[33] Conroy DE, Yang C, Maher JP (2014). Behavior change techniques in top-ranked mobile apps for physical activity. Am J Prev Med.

[34] Kones R (2011). Primary prevention of coronary heart disease: integration of new data, evolving views, revised goals, and role of rosuvastatin in management. A comprehensive survey. Drug Des Devel Ther.

[35] Park LG, Beatty A, Stafford Z, Whooley MA (2016). Mobile phone interventions for the secondary prevention of cardiovascular disease. Prog Cardiovasc Dis.

[36] Penninx BW (2017). Depression and cardiovascular disease: epidemiological evidence on their linking mechanisms. Neurosci Biobehav Rev.

[37] Matthew-Maich N, Harris L, Ploeg J, Markle-Reid M, Valaitis R, Ibrahim S, Gafni A, Isaacs S (2016). Designing, implementing, and evaluating mobile health technologies for managing chronic conditions in older adults: a scoping review. JMIR Mhealth Uhealth.

